# Sex differences in crossover interference in house mice

**DOI:** 10.1101/2025.08.10.669551

**Published:** 2025-08-12

**Authors:** Andrew P Morgan

**Affiliations:** * Department of Medicine, University of North Carolina, Chapel Hill, NC 27514, USA

**Keywords:** meiosis, crossover interference

## Abstract

Meiotic recombination ensures the fidelity of chromosome segregation in most organisms with sexual reproduction. The distribution of crossovers along chromosomes is governed in part by interference, which prevents multiple crossovers from occurring in close proximity, though not all crossovers are subject to interference. Neither the factors that control strength of interference, nor the extent to which they vary within and between species, are well understood. Here we confirm that that crossover interference is stronger in male than in female meiosis in house mice (*Mus musculus*), provide the first estimate of the proportion of non-interfering crossovers in female mice, and show that this proportion is lower than in males. Interference is stronger on shorter chromosomes in both sexes, but the frequency of interference escape is similar across the range of chromosome size. Together with evidence that interference varies across strains and subspecies, our results provide a foundation for studying the evolution and sexual dimorphism in this important feature of meiosis in mice.

## Introduction

Meiotic recombination is essential for faithful chromosome segregation in most sexually-reproducing organisms (Hassold and Hunt 2001). Both the number and the spatial distribution of crossovers are tightly regulated, but vary within and between species. At fine scale, the position of crossovers is determined in part by the location of double-strand breaks, which in turn are associated with specific sequence motifs and/or epigenetic marks which differ by taxa (reviewed in Paigen and Petkov (2010)). At the chromosome scale, crossovers tend to be spaced more evenly than expected by chance. This observation dates back to the first linkage maps inferred from *Drosophila* (Sturtevant 1913, 1915) and is known as crossover interference.

The mechanisms and evolutionary significance of crossover interference remain incompletely understood (Hunter 2015). Muller (1916) first speculated that even spacing of crossovers might promote orderly chromosome segregation. There is now ample evidence that aberrant placement of crossovers with respect to the centromere, telomere or to each other contributes to nondisjunction (Hassold et al. 1991; Koehler et al. 1996; Hassold and Hunt 2001). Others (Kleckner et al. 2004; Wang et al. 2015) have proposed that interference arises as a consequence of processes that ensure at least one crossover per bivalent (Jones 1984) and maintain the total number of crossovers per meiosis within some optimum range (Martini et al. 2006; Cole et al. 2012). Further, a subset of crossovers are also processed via a distinct pathway that is not subject to interference (Santos et al. 2003; Higgins et al. 2004; Guillon et al. 2005; Holloway et al. 2008). Like other features of the recombination landscape (Morelli and Cohen 2005; Sardell and Kirkpatrick 2020)), both the magnitude of interference and the frequency of interference escape vary between sexes in several mammal species (Otto and Payseur 2019). Comparative studies are hindered by the relative paucity of empirical estimates of interference parameters in both sexes. Recombination tends to be much easier to study in the male than in the female germline – spermatocytes are usually more readily obtained than oocytes, and breeding many progeny from one male is often less resource-intensive than breeding the same number of progeny from one female. Our knowledge of meiosis in mammals is thus biased towards the male germline.

Here we use a large panel of 8-way intercross pedigrees from the Collaborative Cross (CC) (Churchill et al. 2004) to provide sex-specific estimates of the strength of crossover interference and the proportion of non-interfering crossovers in both sexes of house mice (*Mus musculus*). The CC is an excellent vehicle for studying recombination (Liu et al. 2014) because it comprises hundreds of informative meioses through both sexes in a randomized genetic background with balanced contributions from 8 founder strains representing all the major subspecies of mice (*M. m. domesticus*, *M. m. musculus* and *M. m. castaneus*). We confirm that interference is stronger in males than females and show for the first time that the proportion of non-interfering crossovers is also greater in males than females. Longer chromosomes show stronger interference than shorter chromosomes in both sexes, but no difference in proportion of non-interfering crossovers.

## Results and discussion

**Figure 1:**
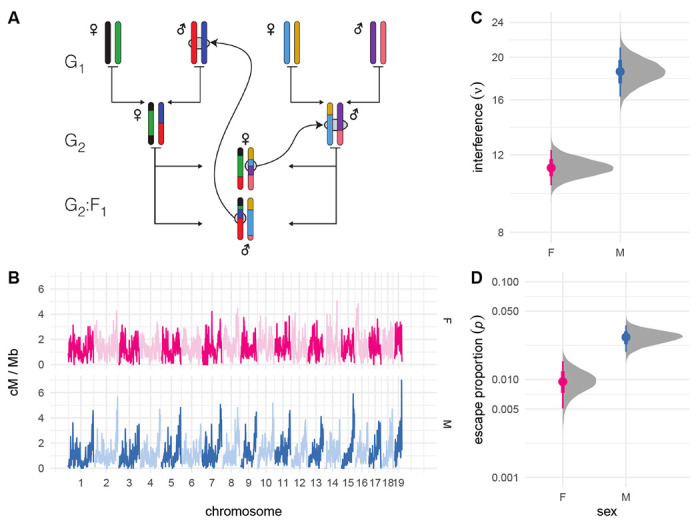
Estimating crossover interference genome-wide. (**A**) Pedigree of a representative intercross family. Founder haplotypes are color-coded, and each family is uniquely defined by the order of founder strains in the first generation. Crossovers identified in the G_2_:F_1_ offspring can be assigned to exactly one meiosis. (**B**) Sex-specific genetic maps, smoothed over 1 Mb windows. (**C,D**) Posterior distribution of global sex-specific estimates of *ν* and *p*. Solid dots show posterior mean; bars show 25% – 75%ile (thick) and 2.5% – 97.5% (thin) quantile intervals.

We obtained fully phased autosomal haplotypes from one male and one female offspring from each of 237 intercross families with the structure shown in Figure 1A. Briefly, the 8 founder strains (G_0_ generation, not shown) were randomly intercrossed to create the G_1_ hybrids, which were again intercrossed to create the G_2_ hybrids. The offspring of the G_2_ pairs are denoted the G_2_:F_1_ and their genomes are a readout of the meioses in the G_1_ and G_2_ animals. Pedigree constraints allow each haplotype junction to be assigned to a crossover event in exactly one of 8 meiosis, so the 474 offspring provide at least partial information for the outcome of 1 896 meioses (see Liu et al. (2014)). (Crossovers in the G_1_ meioses are only observed if they are transmitted to the G_2_:F_1_.) We limit our attention to the 948 fully observed meioses in the G_2_ generation, which resulted in a total of 12 191 crossovers (6 537 in female and 5 654 in male meioses). Of 18 012 transmitted chromosomes, 7 457 (41.4%) were non-recombinant, 8 950 (49.7%) had exactly one crossover and 1 605 (8.9%) had two or more crossovers. An average of 13.8 crossovers were transmitted per meiosis in females and 11.9 in males. On chromosomes with more than crossover, mean distance between adjacent crossovers was 44.8 cM (SD 14.2 cM) in females and 44.4 cM (SD 13.0 cM) in maless, on the sex-specific genetic maps estimated from this population (Figure 1B).

Sex-specific interference parameters were estimated under the “gammma-sprinkling” model of crossover interference (Housworth and Stahl 2003) using an extension of a Bayesian procedure we have described previously (Morgan and Payseur 2024) (Figure 1C,D). Interference was 1.6 times stronger in males (*ν*_male_ = 18.6 [95% HPDI 16.3 – 21.1]) than in females (*ν*_female_ = 11.2 [95% HPDI 10.2 – 12.3]). The proportion of crossovers that escape interference was also 2.9 times greater in males (*p*_male_ = 0.027 [95% HPDI 0.019 – 0.036]) than in females (*p*_female_ = 0.0094 [95% HPDI 0.0054 – 0.016]).

**Figure 2:**
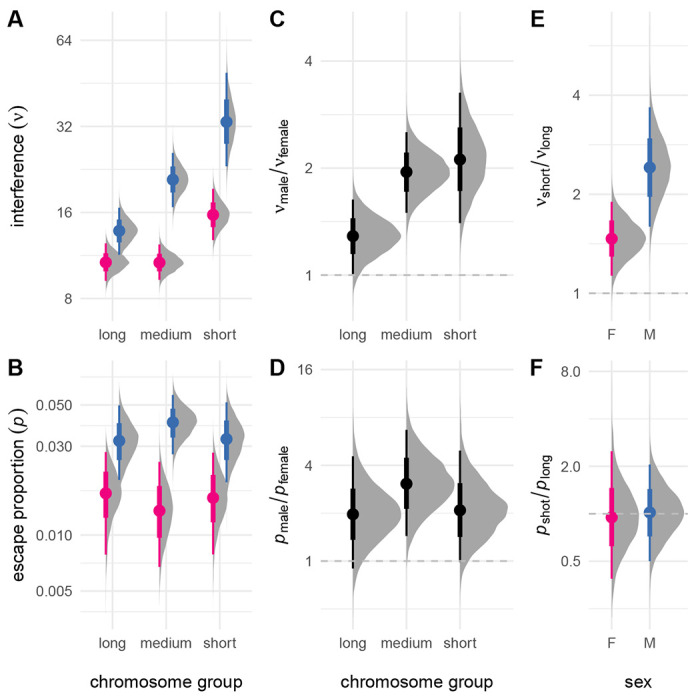
Interchromosomal variation in crossover interference. (**A**) Strength of interference estimated for chromosomes binned by size (“long”, chromosomes 1-5; “medium”, chromosomes 6-12; “short”, chromosomes 13-19). (**B,C**) Proportion of non-interfering crossovers. (**C**) Sex contrasts for *ν* and *p*, expressed as ratios. (**D,E**) Sex-specific contrasts between *ν* and *p* for “short” and “long” chromosomes.

Many prior studies have shown that crossover interference is heterogeneous across chromosomes. We extended our model to allow chromosome-specific values of *ν* and *p* but were not able to obtain stable estimates owing to limited sample size. Results from a reduced model without interference escape were more stable and showed strong negative correlation between *ν* and chromosome length in both sexes (Spearman’s *ρ* = −0.39, *p* = 0.017; Figure S1). We reasoned that we could at least partially capture this signal by aggregating over chromosomes of similar size, and therefore divided the them into three groups, each comprising about one-third of the cumulative genetic map. Results are shown in Figure 2. Interference was clearly stronger on shorter relative to longer chromosomes – 1.5 times (95% HPDI 1.1 – 1.9) stronger in females and 2.4 times (95% HPDI 1.6 – 3.7) stronger in males. The proportion of non-interfering crossovers was similar across the range of chromosome size (just 0.96 times greater in females and 1.0 times greater in males, not significantly different from unity). Sex differences in interference parameters were preserved across the range of chromosome size, though the difference in strength of interference between short and long chromosomes was greater in males. Considering female meioses only, interference on the X chromosome was similar in magnitude to that on autosomes of similar size (Figure S2).

Previous studies in mice based on linkage mapping of chromosome 1 (Petkov et al. 2007) and cytological analyses (de Boer et al. 2006; Peterson and Payseur 2021) have shown stronger interference in males than females. We confirm those findings. Our estimate of *ν*_female_ = 11.2 was nearly identical to the only previous estimate from linkage data (11.3) in (*Mus musculus* × *Mus spretus*) F_1_ females (Broman et al. 2002). As with cattle (Wang et al. 2016), pigs (Brekke et al. 2025), domestic dogs (Campbell et al. 2016) and humans (Campbell et al. 2015), we observed an inverse relationship between (genetic) chromosome size and chromosome-level interference in both sexes. This results in a negative correlation between the genome-wide strength of interference and the average genetic length of a chromosome arm across species (Spearman’s *ρ* = −0.66, *p* = 0.022; Figure S3), though there is no such relationship with the proportion of non-interfering crossovers (Spearman’s *ρ* = 0.28, *p* = 0.38). The proportion of non-interfering crossovers in mice was about half as large as in these other species (Table 1). At least in this small group of species, there is no consistent pattern as to whether interference is stronger in males or females; in the sex with the longer or shorter genetic map; or in the sex with more or fewer non-interfering crossovers.

Why does crossover interference differ between sexes? One possibility is that the physical process(es) that constitute interference, whatever they are, scale with the length of the synaptonemal complex (SC). The SC is longer in females in humans (Tease and Hultén 2004) and mice (de Boer et al. 2006), and crossover counts are positively correlated with SC length within and across individuals in many species (Lynn et al. 2002; de Boer et al. 2006; Dumont and Payseur 2011; Wang et al. 2019a,b). Petkov et al. (2007) showed that the coefficient of coincidence, a measure of interference between pairs of loci, depends on distance between loci on the SC (in μm) rather than in genomic coordinates (base pairs) in mice. This predicts that interference will be stronger in the sex with shorter SC relative to genome size and shorter genetic map. Humans, mice and pigs fit this pattern; dogs and cows do not. The relationship between SC length and recombination rate is very well-conserved while the direction of sex differences in interference is not. Factors beyond SC length must therefore be at play and it seems likely that some of these are lineage-specific. Since crossovers tend to be suppressed near centromeres (Hunter 2015) but the number of crossovers per meiosis is proportional to the number of chromosome arms (Pardo-Manuel de Villena and Sapienza 2001), lineage-specific features of chromosome organization and karyotype may contribute. So too may differences in life history: longer generation time means longer duration of meiotic arrest, and may favor weaker interference in females for optimal positioning of crossovers for secure attachment to the spindle (Kong et al. 2004; Campbell et al. 2015). It is tempting to speculate that sex differences in crossover interference are related to the unique susceptibility of the female germline to meiotic drive – to the extent that weaker interference alters the probability of a crossover between the centromere and a driving locus, it could oppose the spread of the driver if it acts at meiosis I, or favor spread if it acts at meiosis II (Brandvain and Coop 2012). The direction and magnitude of effect would depend on the genetic position, mechanism of action, and fitness effect of the driving allele. Finally, sex differences in interference may arise indirectly from the balance between sex-specific (and possibly sexually-antagonistic) selective pressures on recombination rate (Mank 2009; Dumont 2017) and mechanisms that ensure crossover homeostasis.

What explains the divergent relationship between *ν* and *p* on chromosome size? The biophysical constraints on the maximum number of crossovers and minimum spacing of crossovers compatible with successful chromosome segregation, may be such that chromosomes below some threshold size can accommodate a one and only one crossover. That corresponds to complete interference (*ν* → ∞), and indeed, small chromosomes tend to have larger values of *ν* across species. If the non-interfering pathway is not be subject to the same constraints, we predict that *p* would be uncorrelated with chromosome size.

Some caveats do apply to our results. First, by using haplotypes transmitted in live offspring as the substrate for analysis, we limit our study to viable gametes only. To the extent that interference is systematically different in gametes which are eliminated by meiotic checkpoints or cannot produce a viable zygote, our findings may be biased. Second, some caution is required when extending conclusions about interference and interference escape as statistical phenomena to the underlying molecular processes. In particular, though we infer the proportion of crossovers that escape interference in aggregate (*p*), we have no way of designating specific crossovers as non-interfering. The gamma-sprinkling model generally has better fit to empirical data than simpler models (Otto and Payseur 2019), but the relationship between *p* and the proportion of crossovers molecularly defined as non-interfering still needs to be validated.

## Materials and methods

This work re-analyzes published crossover data from the mouse Collaborative Cross (CC) project (Churchill et al. 2004). Briefly, the CC is a panel of recombinant inbred mouse lines (RILs), each of which was initiated with an 8-way intercross followed by inbreeding. One male and one female of the last outbreeding generation in a subset of 237 lines were genotyped using a high-density SNP array and used to characterize the genome of the incipient RILs. Mouse breeding, DNA extraction, genotyping, haplotype inference, and construction of the recombination map are described in detail in Liu et al. (2014). Coordinates of haplotype segments were defined on the sex-specific genetic map estimated from this cross.

Crossover interference was analyzed under the “gamma-sprinkling” (or Housworth-Stahl) model. Some proportion *p* ∈ (0, 1) of crossovers are modeled as non-interfering, while the remaining 1 – *p* are subject to interference under the gamma model (Broman and Weber 2000) in which the strength of interference is represented by the single unitless parameter *ν* > 0. (Note that our parameterization assumes that *p* is strictly positive, though it can be arbitrarily small. This differs from some other implementations.) We allow both *ν* and *p* to vary across groups of meioses (indexed by *i* = 1, ‖ *m*) and groups of chromosomes (indexed by *j* = 1, … *n*), using an extension of the hiearchical Bayesian approach described in (Morgan and Payseur 2024). Let group-specific parameters *ν_ij_*, *p_ij_* be defined as follows:

(1) $$\text{log}\left(v_{ij}\right)=\beta_{0}+\beta_{i}+\beta_{j}$$

(2) $$\text{logit}\left(p_{ij}\right)=\alpha_{0}+\alpha_{i}+\alpha_{j}$$

where *β*_0_ is a global intercept term (to apply some shrinkage) and the *β_i_* and *β_j_* are subject- and chromosome-specific effects. The prior distribution for $\beta_{0}∼𝒩\left(0,\sigma_{\nu }^{2}\right))$ while $\beta ∼\text{MVN}\left(0,\sigma_{\nu }^{2}I_{m}\right)$ That is, group-specific *β_i_* are assumed uncorrelated. The *α* terms are defined similarly. The likelihood of a set of transmitted chromosome segments, given the parameter vector ***θ*** = (***β**, **α***), was calculated with the C routine R_stahl_loglik from the R package xoi v0.72 (Broman 2023). Inference was done by a simple Metropolis-coupled Markov chain Monte Carlo (MCMC) sampler, with proposal distribution tuned to achieve a rejection rate of around 0.4. The sampler was run for 25 000 iterations, of which the first 5 000 were discarded as burn-in. Quantities of interest were calculated directly from the posterior distributions, with summary statistics calculated using R package coda v0.19-4.1.

## Supplementary Material

Supplement 1

2

## Figures and Tables

**Table 1:** Sex-specific interference parameters for available mammal species.

species	sex	cM	N	FN	*v*	p	reference
mouse	M	1221	19	19	18.60	0.0268	this study
	F	1355			11.20	0.009 37	
dog	M	1816	38	38	14.05	0.055	Campbell et al. (2016)
	F	2162			30.64	0.035	
cow (Holstein)	M	2519	29	58	8.70	0.0834	Wang et al. (2016)
	F	2372			9.96	0.1002	
cow (Jersey)	M	2369	29	58	6.70	0.0220	Wang et al. (2016)
	F	2229			7.55	0.0289	
pig	M	1915	18	32	8.54	0.049	Brekke et al. (2025)
	F	2664			6.45	0.043	
human	M	2708	23	46	8.93	0.067	Campbell et al. (2015)
	F	4355			7.19	0.078	

cM, length of the autosomal recombination map. N, haploid chromosome number. FN, number of haploid chromosome arms (“fundamental number”, (Matthey 1945)).

## Data Availability

Processed data underlying key analyses in this manuscript will be available on Figshare: doi:10.6084/m9.figshare.29876591 Analysis code is available on Github: https://github.com/andrewparkermorgan/mouse_crossover_interference_2
